# Health Behavior Survey Among People Who Use Opioids: Protocol for Implementing Technology-Based Rapid Response Surveillance in Community Settings

**DOI:** 10.2196/25575

**Published:** 2021-09-10

**Authors:** Paula M Frew, Laura A Randall, Adrian R King, Jay T Schamel, Anne C Spaulding, HBOU Study Team, Ian W Holloway

**Affiliations:** 1 University of Nevada Las Vegas School of Public Health Las Vegas, NV United States; 2 UNLV Population Health & Health Equity Initiative Las Vegas, NV United States; 3 Department of Epidemiology Emory University School of Public Health Atlanta, GA United States; 4 Department of Social Welfare UCLA Luskin School of Public Affairs Los Angeles, CA United States

**Keywords:** substance use, opioid, opioid crisis, social determinants, hidden populations, health equity

## Abstract

**Background:**

In 2018, 2 million Americans met the Diagnostic and Statistical Manual of Mental Disorders, 5th Edition diagnostic criteria for an opioid use disorder, and 9.9 million Americans had misused prescription pain relievers the previous year. Despite a rapid increase in opioid misuse, opioid use disorders, and overdoses, data are limited on the behavioral and contextual risks as well as the protective factors fueling the opioid epidemic in some hard hit US cities—Atlanta, Los Angeles, and Las Vegas. Opioid use also contributes to the risk of other health problems such as HIV and hepatitis C virus infections or mental health disorders and is linked to behavioral and environmental risks (eg, homelessness, experiences of violence, involvement in the justice system). Knowledge of the relationships between these linked vulnerabilities and how they influence service utilization is critical to effective policy and interventions.

**Objective:**

This survey explores the relationships between demographic and economic characteristics, behavioral and environmental risk factors, and service utilization of people who use opioids to inform public health practice, policy, and future efforts to mitigate the risks faced by this population experiencing multiple health, social, and economic vulnerabilities. The results of this survey will be used to identify needs and intervention points for people who use drugs currently served by public health organizations.

**Methods:**

We implemented a community-engaged strategy that involved development and execution of a two-stage purposive sampling plan involving selection of partner organizations (syringe exchange programs in urban settings) and recruitment and enrollment of participants aged 18-69 years served by these organizations in Atlanta, Los Angeles, and Las Vegas from 2019 to 2020. The recruited participants completed a survey, including a variety of measures to assess health (physical and mental) and health behaviors such as sexual behavior, vaccine receipt, and HIV/ hepatitis C virus infection testing. Additional items assessed drug use and misuse, syringe exchange and health service utilization, sex exchange, histories of interpersonal violence, and vaccine confidence.

**Results:**

This protocol was successfully implemented despite challenges such as real-time technology issues and rapidly finding and surveying a difficult-to-reach population. We sampled 1127 unique participants (248 in Atlanta, 465 in Los Angeles, and 414 in Las Vegas).

**Conclusions:**

The establishment and utilization of strong community partnerships enabled the rapid collection of data from a typically difficult-to-reach population. Local efforts such as these are needed to develop policies and practices that promote harm reduction among people who use opioids.

**International Registered Report Identifier (IRRID):**

RR1-10.2196/25575

## Introduction

Modifications in use and prescription practices of opioids in the late 1990s caused a major shift in the mindset toward opioids among the general public as well as among health care professionals [1]. Aggressive marketing campaigns from pharmaceutical companies created an increase in the number of prescriptions written to combat noncancer pain in patients. As an October 2020 settlement with the United States Department of Justice stipulates, the industry adopted practices that led to a rapid proliferation of abuse of both prescription and nonprescription opioids.

According to the National Survey on Drug Use and Health, over 10 million people in the United States aged 12 years or older (roughly 4% of the total population) misused opioids in 2018 [2]. Nearly 10 million of these individuals reported prescription pain reliever misuse, and 808,000 reported using heroin [2]. That same year, an estimated 2 million people aged 12 years or older had an opioid use disorder (less than 1% of the population) [2]. However, among those who met the criteria for an opioid use disorder, only about 400,000 (19.7%) received drug use treatment at a specialty facility in the past year (eg, inpatient hospital, inpatient or outpatient drug or alcohol rehabilitation facility, inpatient or outpatient mental health center) [2].

Synthetic opioids have become a major contributor to the significant increase in opioid-related overdose deaths in the United States [3]. The United States Centers for Disease Control and Prevention reported that more than 31,000 fentanyl-related fatalities occurred in the United States in 2018, accounting for the most deaths among any type of opioids [4]. More specifically, death rates associated with synthetic opioids increased by 10% from 2017 to 2018 [4]. Other data suggest that many people who inject drugs were unaware or suspect that their drugs contain fentanyl more often than not [5,6], thereby exponentially increasing their risk of opioid-related overdose and death. Furthermore, data from 2018 estimated that, on a daily basis, 128 Americans die as a result of opioid-related overdoses, which reiterates a serious national and public health concern [7].

The impact of the opioid epidemic in the United States on public health extends beyond opioid-related fatalities. Additional public health consequences related to opioid misuse and opioid use disorders include an increase in HIV/hepatitis C virus infections and other infections among persons who inject drugs [8-10]. The United States Centers for Disease Control and Prevention reported that, in 2018, 1 in 15 new HIV diagnoses in the United States occurred among persons who inject drugs [11]. Additional concerns included increased risk of neonatal abstinence syndrome and other fetal and birth complications [12], health and safety of children whose parents are active users [13], impact on mental health [14], impact on first responders and other health care professionals [15], and economic costs [16].

More recently, opioid drug overdoses have increased as a result of the COVID-19 pandemic [17]. More than 40 states have reported not only increases in opioid-related mortalities but also concerns for those experiencing mental illness or substance use disorders [17]. According to the Overdose Detection Mapping Application Program, the proportion of suspected overdoses in the United States has jumped from 18% in March 2020 to 42% in May 2020 [18]. Some attribute this rise in overdoses to the directives for social distancing resulting in isolation, while others have noted considerable increases in alcohol and other substance use since the COVID-19 pandemic began [19]. It has also been suggested that impacts to the drug supply have resulted in users turning to new substances or dealers [19].

Given these findings regarding the public health consequences associated with opioid use, it is vital to gain additional information relative to the behavioral and contextual risk and protective factors fueling the current epidemic. Prior studies have identified correlations between opioid abuse and behavioral and environmental risks, including high-risk sexual behavior [20,21], homelessness [22,23], experiences of violence [24], and involvement in the justice system [25]. Further, previous studies have indicated that people who use opioids also display higher rates of serious mental illness [26], which can compound other issues of substance abuse [27]. However, many of these domains are sparse, and studies are often restricted to emergency room patients or are limited in scope and geographic coverage. Additionally, to our knowledge, no prior research has estimated vaccination coverage or confidence among people who use opioids. Strategies focused on harm reduction will not only inform public health practice and policy but also mitigate the risks faced by vulnerable populations. Our project has been developed to expand our knowledge of these behavioral and contextual risks and to address our limited knowledge of these barriers and intervention points.

Through the development and maintenance of strong partnerships with community organizations serving people who use opioids, we were able to work with client populations that are normally difficult to engage in data collection efforts related to their health behaviors and drug use. To characterize the health status and behaviors of people who use opioids and utilize services at community organizations, we partnered with community organizations providing syringe exchange services in 3 US cities to recruit people who use opioids to participate in a comprehensive health and behavior questionnaire, which was designed to capture the following project goals:

1. Assess the behavioral (drug use patterns, vaccination status, pre-exposure prophylaxis use, sexual behavior, etc) and environmental (homelessness, experiences with violence, etc) health risks faced by people who use opioids who are clients of community health organizations.
2. Examine barriers and facilitators of access to health and life services, such as financial support, insurance, access to transportation, and social support networks.
3. Determine utilization of health and life services from the partner community health organizations and from other sources to identify service gaps that could be targeted by interventions and community organizations.

The results of these analyses will be used to identify needs and intervention points for people who use opioids who use services at community health organizations and to help inform service provision and policy.

## Methods

### Site and Partnering Organization Selection

The sampling strategy included a recruitment plan of up to 1200 individuals who identified as clients of partnering organizations. Data collection sites were chosen based on characteristics of the population of people using opioids, particularly those who may be vulnerable to HIV, viral hepatitis, vaccine-preventable diseases, and opioid use. The selected cities were Atlanta, Georgia; Los Angeles, California; and Las Vegas, Nevada. We engaged partners with whom the project leadership team has spent several years cultivating collaborative relationships. These partnerships have emerged from the project faculty reaching out to organizations to gauge interest in partnering on various field-based efforts related to service provision, health outcomes, and public policies that impact their client populations. The partners engaged in our efforts had well-established syringe services and a demonstrated track record of success serving diverse clients in a variety of settings. Atlanta’s primary partner organization provides syringe services through a mobile program, with established locations in the community where individuals can come on set days/times, as well as community outreach at homeless encampments and other known drug use locations. Our primary Los Angeles partner is a community-based health care and social services organization working to identify and address emerging health issues faced by the Latino and lesbian, gay, bisexual, transgender, and queer (LGBTQ+) communities. They provide syringe services at one of their brick-and-mortar sites as well as through a mobile program. The Las Vegas partner provides services at a brick-and-mortar site and through a mobile program, as well as through local vending machines and delivering materials to their clients’ homes via FedEx. The organization aims to reduce the risk of negative behavioral consequences while eliminating the stigma associated with drug use. These community partners were selected in part for variation in operations, as this would provide for a better assessment of the differences in their operations and their ability to provide services. As needed, we supplemented our sample with participants recruited from similar entities servicing these communities; in Atlanta, we supplemented with another organization, and in Los Angeles, we supplemented with 2 additional agencies.

### Participant Inclusion and Exclusion Criteria

Eligible participants included those who had accessed the designated partner’s services. Potential participants reported using opioids at least once in the previous 6 months, being 18-69 years of age, having English or Spanish language comprehension (reading or speaking), and were able to voluntarily consent to participation. Exclusion criteria were inability to provide voluntary consent (for any reason) and failure to meet other inclusion criteria. Pregnant women were not a target population for this survey project; yet, they were not explicitly excluded if they met all the inclusion criteria.

### Sampling Plan and Sample Size

We used a two-stage purposive sampling plan: (1) selection of the partner organizations and (2) recruitment and enrollment of clients utilizing services from these entities. Sites were selected based on being operational for at least two years and having a variety of service delivery modes (eg, entirely mobile, combination brick and mortar/mobile services). Recruitment and enrollment of participating individuals were based on the eligibility criteria and sampling frame. Purposive sampling is based on having strong theoretical reasons for inclusion of cases in the sample. In contrast to probabilistic methods, purposive sampling draws on theory and empirical data to select the most information-rich cases to inform the project aims in question while having sufficient variation to allow for analytic comparisons [17]. Variations for this project included selected partner sites, each of which operates under different state-based regulations and differs in service provision. We also sought to have varied distribution by race, ethnicity, and gender identity across the 3 sites. The planned variation by these characteristics was based on anecdotal data about the differences in this population’s lived experiences and perceived needs, barriers, and facilitators to accessing services. We were also mindful of capturing a spectrum of ages across the eligible 18-69 years of age range. A sampling target of 400 per city (total of 1200) was chosen to fit within funding restraints while providing an adequate sample size for city-specific and comparative analyses. This sampling target also ensured adequate samples of subpopulations such as LGBTQ+ gender identity.

### Staff Training

Project staff were recruited to the team by the University of Nevada, Las Vegas and the University of California, Los Angeles project leaders, including community members, students, and young professionals who expressed interest in the topic area and had demonstrated exemplary skills, subject matter knowledge, and experience in similar roles with the populations of interest. At the outset of the training process, all field staff were required to complete Collaborative Institutional Training Initiative trainings related to ethics and procedures. Survey-specific training included a full-day review of best practices in survey methods as well as methods for interacting with at-risk socially and economically disadvantaged populations. The staff discussed appropriate methods for selecting participants in field settings and team-based assessment procedures to ensure that potential participants were not currently under the influence of impairing drugs or alcohol prior to consent. The training detailed the manner in which partner staff were provided an opportunity to identify potential participants, given their experience and knowledge of clients in the local population. During the training events in each selected city, the team also took time to navigate the survey on the tablet to become comfortable with the format and to identify strategies to address potential issues that participants might experience. As diverse staff were hired to conduct the survey, including those bilingual in English and Spanish, project leaders spent considerable time during training to ensure that survey procedures, instructions, and domain items were clear to all team members by using both languages prior to field initiation. This process also allowed the team to check survey skip and display logic to ensure proper survey flow and item interpretation. Quality assurance procedures were also identified and discussed in the one-day trainings, including details on potential mitigation strategies. All project staff conducting surveys, as well as its team leaders in each city, participated in face-to-face and follow-up trainings on the instruments and procedures, including a review of acceptable strategies to approach potential participants for data collection.

### Field Recruitment Process

The partner organizations assisted in identifying the best approaches for recruitment of their clients, scheduling data collection periods, and performing data collection in diverse field settings (eg, during mobile outreach services). Participating partners designated point-of-contact community-based organization (CBO) staff who could answer questions about the project and redirect those interested in speaking to designated project recruiters as needed. Potentially eligible participants were screened in-person at specific locations during data collection events. All recruiters assessed eligibility via the established electronic tablet-based screener. The screening tool allowed the team to collect data confidentially as no identifying information was needed from participants and provided us the opportunity to collect consent electronically without collecting the name or signature on consent documents. In addition to determining eligibility, the screening process also included basic demographic questions (eg, age, race/ethnicity). Screening took no more than 3 minutes to complete.

### Informed Consent

Survey procedures involved a two-stage consent process. Initial consent, conducted at the beginning of the screening process, took place before any questions were asked. This initial abridged consent described the project aims, including the length of time required for participation, the participants’ rights not to answer any question(s) they did not wish to answer, the right to leave at any time, and notice that the information they provided would be destroyed if they chose not to participate, and concluded by asking for their consent to proceed. The initial consent was used because the screeners included eligibility as well as demographic questions. These data were linked via a unique identification number to the survey; thus, we sought to ensure that participants understood and accepted this process prior to answering any questions. At the end of the screening process, the survey automatically directed eligible participants to our web-based informed consent form where participants provided consent and were able to continue to the rest of the survey questions. The web-based consent process reiterated the information previously shared during the initial consent. During both consent processes, participants were given an opportunity to ask questions and receive additional information before providing consent. The reading level for the electronic consent was no higher than an eighth-grade level on the Flesch-Kincaid reading scale. Participants were recruited and surveyed at sites where they already received services of value to their health. At each location, participants were able to take a nominal well-being item typically available at the sites for clients (eg, hand sanitizer, hygiene kit, sunscreen).

### Data Collection Instruments

In addition to the informed consent form and screener, data were collected using a Qualtrics-based questionnaire on a Wi-Fi–enabled iPad. A high-quality data collection instrument was developed to address the constructs of interest, maximize participant engagement, minimize missing data, and minimize participant burden. These goals guided the development of the screener and the survey. Program staff from participating organizations and other subject matter experts provided feedback on the survey which the team used to refine survey questions. The survey took approximately 20-30 minutes to complete (median 23 minutes, IQR 17-31 minutes). Our survey instruments in English and Spanish asked about participant sociodemographic data, health behaviors (including sexual behavior, vaccination, HIV and hepatitis C virus testing), health literacy, co-occurring psychiatric symptoms, legal issues (including knowledge of Good Samaritan laws and incarceration history), history of traumatic experiences, social networks, drug and alcohol usage, media use, intervention access, and CBO service utilization. A description of the content domains and example items are available in Multimedia Appendix 1 (Table S1). Psychometric instruments were drawn from valid and reliable measures: vaccine confidence was assessed using a version of the Emory Vaccine Confidence Index modified for adult vaccination [28], mental distress was measured using the K6 scale [29], health literacy assessment used the 3-item scale of Chew et al [30], and the DUDIT-E instrument was used to measure the motivation to change substance abuse behavior [31]. Other items were drawn from prior surveys when available and from subject area expertise.

### Technology Adoption and Data Collection Procedures

Participants were identified by an identification number only. We obtained demographic information during the screener and survey, which was collected on a Health Insurance Portability and Accountability Act (HIPAA)–compliant survey administration platform, that is, Qualtrics (Provo, UT). HITECH (Health Information Technology for Economic and Clinical Health Act) updated HIPAA rules to ensure that data were properly protected and the best security practices followed. Qualtrics safeguards all data and uses secure data centers to ensure the highest protection per HITECH requirements.

### Data Analysis

The protocol data analysis plans include univariate and multivariate statistical techniques to understand factors related to opioid use as well as perceived needs related to HIV/hepatitis C virus infections and other partner agency services among people who use opioids in and across the 3 cities. Planned analyses include assessments of the following:

1. Vaccination coverage and vaccine confidence.
2. Acceptability of injectable pre-exposure prophylaxis among people who inject drugs.
3. Prevalence of mental distress and its association with violence and victimization.
4. Knowledge of and attitudes toward Good Samaritan laws.
5. Current CBO service and other health services utilization, desired services, and potential barriers to service utilization.
6. Patterns of substance use, including alcohol and tobacco, and attitudes toward substance abuse.

These assessments will also include comprehensive analyses of the associated risk and protective factors. Estimates of bivariate associations will be used to describe population-level trends, while appropriate multivariable models will allow adjustment for relationships between independent factors. Data analyses associated with specific survey construct domains are described in Table S1 of Multimedia Appendix 1.

### Institutional Review Board Approval

Project protocols were reviewed and given an exempt determination by the University of Nevada, Las Vegas Institutional Review Board, followed by independent institutional review board reviews with Emory University, University of California, Los Angeles, and College of Mount Saint Vincent for secondary analyses determination.

## Results

Through data collection, 1368 individual surveys were started across the 3 sites from May 2019 through February 2020. The majority (1127/1368, 82.4%) of the participants who started the survey completed it and met our eligibility criteria. In total, 1127 participants met the eligibility criteria and completed the survey (Atlanta, n=248; Los Angeles, n=465; Las Vegas, n=414).

## Discussion

### Procedural Advantages of Technology Adoption

Our use of tablets allowed us to strengthen our recruitment efforts for opioid users who may have variable transience among the population and be harder to reach. Utilizing the tablets allowed our team members to conduct recruitment while in brick-and-mortar partner agency locations and participate in mobile outreach, which occurred in all 3 of our sites. Our use of technology also allowed our recruiters to troubleshoot issues in survey completion while participants self-administered the survey. For example, some participants had issues with visual impairment, making use of the electronic tablets difficult to complete the survey in a timely manner; however, we were able to adjust the font size to be easier to read for our participants. Lastly, embracing the web-based Qualtrics platform for survey completion across our 3 sites facilitated immediate review and assessment of collected data and planning for additional data collection needs. Project staff were able to immediately examine patterns in data collection across the 3 sites to identify effective recruitment strategies and modalities in each site (eg, brick-and-mortar compared to mobile outreach). We were also able to identify any potentially problematic questions within the survey and to assess response patterns across the 3 sites to identify participants simply skipping through the survey. Tracking of our participants through web-based channels and close communication with CBO staff members also ensured that no individual participant completed the survey more than once.

### Limitations

This effort focused on people who use opioids and reside in 3 cities highly impacted by the US opioid crisis and who receive services through the partnering organizations. We excluded other groups who were known to underutilize HIV, hepatitis C virus, and other prevention and treatment services, such as individuals of 13-17 years of age and people who do not use opioids. Despite efforts to develop an instrument with health literacy in mind (eg, Flesch-Kincaid lower than the eighth-grade reading level), we experienced some item response gaps as some participants were unable to respond to some questions without excessive clarification or direction from staff and were therefore unable to provide complete survey responses. We also encountered a handful of participants who were too inexperienced with technology and could not successfully complete the survey. Finally, across sites, there were other competing ongoing research efforts among the target population, often with higher or more desirable incentives for participation.

### Conclusion

We successfully implemented our web-based, tablet-based survey of opioid users across 3 urban sites by enrolling over 1300 unique client participants. Based on our eligibility framework, over 1100 quality answers remained for use during data analysis. Our project was able to successfully navigate collaboration with local agencies to facilitate participant recruitment. Forging relationships with each of the collaborating entities ensured our successful implementation of the survey across our 3 sites while navigating the needs for recruitment in diverse settings (eg, brick-and-mortar facilities, mobile outreach). We avoided any data duplication from participants completing the survey more than once. Our project demonstrated that recruitment of opioid users in these 3 cities is possible by using appropriate methodology by reaching out to this population in environments where they are comfortable, instead of clinical settings. We believe investigators need to understand the participant population and meet them where they are—not only the physical location but also the provision of a survey that is accessible to the various needs of this population. Lessons learned for future in-person data collection with opioid-using populations in these cities include considering staff-facilitated administration of surveys to ensure comprehension of the survey questions, thereby reducing the time needed for survey completion. Additional technological advances could aid in the implementation of the survey. Specifically, because some participants identified low experience with technology (iPad) and limited literacy level or visual impairments, the capability of the survey to automatically be read to the participant, if they choose, could have been immensely useful among this population. Considering these adaptations when developing and designing future research projects focused on this population would facilitate efficient data collection and capture of quality data.

## Appendix

Multimedia Appendix 1 Content domains and example items and data analyses associated with specific survey construct domains.

## Abbreviations

CBO: community-based organization
HIPAA: Health Insurance Portability and Accountability Act
HITECH: Health Information Technology for Economic and Clinical Health Act
LGBTQ: lesbian, gay, bisexual, transgender, and queer
