# Supplementary material for: Health Behavior Survey Among People Who Use Opioids: Protocol for Implementing Technology-Based Rapid Response Surveillance in Community Settings
Source: JMIR Res Protoc. 2021 Sep 10;10(9):e25575. doi: 10.2196/25575 (PMC8463945; doi:10.2196/25575)
Supplement: Multimedia Appendix 1 [file resprot_v10i9e25575_app1.doc]

**Table S1.** Survey domains, example items, and analyses.

| **Construct** | **Example Items** | **Analyses Plans** |
| --- | --- | --- |
| Demographics and Socioeconomics | - *Age* - *Ethnicity / Race* - *Gender Identity* - *Marital Status* - *Education* - *Employment Status* | - Descriptive analysis of characteristics of sample population. - Used in other analyses for stratification and associations with measures of interest. |
| Self-reported Vaccination Status | - *Below is a list of common vaccines, also known as immunizations. Of these, which vaccine(s) have you had?* | - Estimate vaccine coverage rates and patterns. - Identify demographic associations and identify barriers, facilitators, and predictors of coverage. - Explore strategies for improving vaccination coverage. |
| Vaccine Confidence | Drawn from the eight-item Emory Vaccine Confidence Index.  *Examples: (5-point scale; strongly disagree to strongly agree)*   - *It is important for everyone to get the recommended vaccines for adults.* - *Vaccines recommended for adults are safe.* - *My doctor or nurse has my best interest in mind when making vaccine recommendations.* | - Describe vaccine confidence rates and their correlation with vaccination receipt. - Use bivariate and multivariate models to look for correlates of vaccine confidence, including demographics, education, socioeconomic status, and health literacy. |
| Health Status and Experiences with Healthcare | - *In your lifetime, how many days have you spent hospitalized for a medical problem?* - *Do you have any chronic medical problems (Examples: Diabetes, Obesity, High Blood Pressure, High Cholesterol, etc.) which continue to interfere with your life?* | - Assess prevalence of chronic medical conditions and health care utilization. - Determine barriers to access to health care utilization, and association with the desire for medical services (or linkage to medical services) to be provided by SSPs. |
| Mental Health / Experiences of Mental Illness | - *Have you ever felt paranoid (examples: like everyone is out to get you, sabotage you, follow you, or harm you) in your lifetime, the past month, or the past 24 hours?* - *Are you currently receiving mental health services (examples: counseling, inpatient therapy, outpatient therapy, seeing a psychiatrist)?* - *Have you ever taken medication prescribed for you for a psychiatric or mental health condition?* - *K6 screening scale for serious mental illness.* | - Assess prevalence of serious mental illness, and its association with utilization of mental health services. - Examine demographic, economic, and environmental barriers to mental health service utilization. - Explore the association between serious mental illness and other health and behavior outcomes, including medical healthcare utilization, SSP service utilization, experiences of violence, sexual behavior, and patterns of drugs. |
| Personal Relationships | - *Please indicate how you would describe your relationship with the following friends and relatives (Mother, Father, Former Guardian, Brother(s), Sister(s), Sexual Partners, Spouse/Partner, Children, Friends)* | - Describe strength of personal relationships. - Estimate associations between strength of personal relationships and mental and medical health outcomes, drug utilization patterns and attitudes towards drug use, access to health services, etc. |
| Sexual Behaviors | - *In the past 6 months, how many sexual partners have you had?* - *In the past 6 months, how many of your sexual partners were opiate drug users?* | - Describe sexual behaviors. - Explore correlations between risky behavior and demographics, drug use, protective behavior such as PrEP use, and sexual health service utilization. |
| HIV/HCV/STIs | - *Have you ever been tested for HIV?* - *Have you ever been tested for Hepatitis C Virus?* | - Estimate prevalence of known HIV/HCV/STIs. - Estimate prevalence of testing for HIV/HCV/STIs. - Determine treatment status and location of service for known HIV/HCV infections, as well as correlates of service. |
| PrEP Awareness and Use | *How much have you heard about PrEP?*   - *Have you ever taken PrEP?* - *If you wanted to start taking PrEP, would you know how to get it?* | - Estimate PrEP awareness, knowledge, and use patterns. - Assess the acceptability of different PrEP application methods, including injectable PrEP and spermicidal PrEP, within this population, especially among high-risk subpopulation such as MSM with multiple partners. |
| Health Literacy | - *How often do you have someone help you read hospital or medical materials?* - *How often do you have a problem understanding what medical providers tell you about any condition you may be diagnosed with?* | - Estimate health literacy within this population. - Determine associations between health literacy, health beliefs (e.g. vaccination confidence), behavioral risk-taking, and health service utilization. |
| Resource Negotiation | - *Have you ever traded sex for goods, a place to stay, money, or drugs/alcohol?* - *What type of sex work are you involved in?* | - Estimate prevalence, type, and correlates of sex work. - Assess as a risk factor in other analyses (PrEP use, vaccination, etc.) |
| Service Utilization | - *Currently, do you utilize services offered at [this SSP]?* - *What resources have you utilized that are offered at [this SSP] in the last 6 months?* | - Describe current ssp service utilization and potential desired services. - Assess correlates and predictors for service utilization and desired services. - Identify unmet health/economic/life needs and determine potential barriers to utilization. |
| Experiences of Trauma (physical, mental, emotional, sexual) | - *In your lifetime [/past 6 months], have you experienced any emotional violence (verbal abuse, name calling, manipulation, etc.) from anyone else?* - *In your lifetime [/past 6 months], have you experienced any form of physical violence (slapping, hitting, kicking, pushing, etc.) from anyone else?* - *In your lifetime [/past 6 months], have you experienced any form of sexual violence (unwanted touching, forced sexual activity, incest, molestation, etc.) from anyone else?* | - Estimate prevalence of experiences of trauma. - Assess demographic, environmental, and behavioral correlates and risk factors for experiences of trauma within this population. - Examine associations between experiences of trauma, mental distress, and utilization of mental and medical health services. |
| Substance Use (e.g. alcohol, illicit drugs) | - *Of the substances listed below, please indicate which you have used and the primary way you have administered (used) them in the last 6 months. (Heroin, Methadone, Opiates/Analgesics, Barbiturates, Sedatives, Crack Cocaine, Powder Cocaine, Prescription Amphetamines, Street Amphetamines, Cannabis, Hallucinogens, Inhalants, Spice, Bathsalts).* - *Have you ever attended an inpatient (residential) drug/alcohol treatment center, in your lifetime?* - *Drug Use Disorders Identification Test Extended (DUDIT-E) Treatment Readiness Assessment* | - Estimate prevalence and patterns of use for various drug categories, and methods of application. - Examine factors associated with use of categories of drugs and methods of application. - Estimate associations between alcohol and illicit drug use. - Assess participants’ attitudes towards their use of illicit drugs and readiness for treatment, along with history of substance abuse treatment. |
| Tobacco Use | - *What/which tobacco product(s) have you used in your lifetime? (Select all that apply)* - *During the past 6 months, have you tried to stop using tobacco products?* | - Estimate prevalence and patterns of tobacco use, especially among vulnerable subpopulations such as participants identifying as LGBTQ. - Examine associations between tobacco and illicit drug use, and between participants’ attitudes towards their use. |
| Legal | Incarceration history:   - *Have you ever been incarcerated in a prison or jail?*   *In your lifetime, approximately how much time have you spent incarcerated?*  Naloxone and Overdose Prevention:   - *Have you ever assisted someone you felt was experiencing a drug overdose? (If 'no' please explain)* - *Do you carry Naloxone with you on a normal daily basis?* | Incarceration history:   - *Describe experiences of incarceration.* - *Assess as an environmental/risk factor for vaccination, service utilization, experiences of violence, mental health, and other analyses.*   Naloxone and Overdose Prevention:   - *Estimate prevalence of overdose experience and naloxone use and explore predictors of both.* |
| Technology | - *What kind of cell phone do you own? (smart phone with or without data, talk and text only, no phone)* - *What devices do you use to get online? (Desktop computer, laptop, tablet, cell phone, non-cell mobile device, other, I do not use any device to get online)* | - Describe technology access and use, along with predictors and barriers to access and use. - Examine correlations between general technology use, use of technology for health-related purposes, and health literacy. |
